# Tenofovir disoproxil fumarate reduce incidence of HCC development in CHB-patients with compensated cirrhosis

**DOI:** 10.1186/s13027-018-0203-8

**Published:** 2018-08-29

**Authors:** Ping Zhang, Qingli Liu, Mei Yuan, Lina Wang

**Affiliations:** Clinical laboratory, the sixth people’s hospital of Qingdao, Qingdao, 266000 Shandong China

**Keywords:** Tenofovir, Chronic hepatits B, Hepatocellular carcinoma, Cirrhosis, Nucleus(t)ide analogues

## Abstract

**Background:**

The impact of different anti-virus regimens on prognosis of Chronic hepatitis B (CHB) related cirrhosis remains to be explored. We aim to investigate whether CHB-related HCC patients receiving nucleoside analogue regimen or not have a different prognosis.

**Methods:**

Two hundred forty-two CHB-related compensated cirrhosis patients were attributed into groups regarding their anti-virus regimens containing tenofovir disoproxil fumarate (TDF) or not. The results of two groups were reviewed and investigated. The probability of hepatocellular carcinoma (HCC) development among each group were analyzed and compared.

**Results:**

Two hundred forty-two CHB-related compensated cirrhosis patients from 2008 June to 2011 December were included in our study. One hundred twenty-seven patients received anti-virus regimen containing TDF and 115 patients received anti-virus regimen without TDF. Child-Pugh score, alanine aminotransferase (ALT), total bilirubin level, status of hepatitis B e antigen (HBeAg) and serum HBV DNA level were compared between groups. The cumulative probability of HCC development in TDF-contained group were significantly lower than it in non-TDF-contained group (*p* < 0.05). Multi-variant analysis indicated that TDF-containing regimen treatment was significantly associated with lower probability of HCC development, (hazard ratio, 0.18; 95% confidence interval range, 0.07–0.45, *p* < 0.05).

**Conclusion:**

Anti-virus regimen containing TDF benefits for the prognosis of CHB-related liver cirrhosis patients.

## Background

Hepatocellular carcinoma (HCC) is among the most common malignancies of high morbidity and mortality, especially in China [1, 2]. Chinese HCC patients account for the majority of HCC-related mortality in the world [1–4]. Factors associated with the development of HCC include: hepatitis C virus infection, alcholic hepatic diseases, smoking and et al., among which chronic hepatits B (CHB) infection is highly related to HCC development [5–12]. CHB-related liver cirrhosis and HCC patients is predominant Chinese HCC patients, as hepatits B virus (HBV) infection is highly prevalent in China [13, 14].

Serum HBV-DNA level is a key predictor for liver cirrhosis and it has been proved to correlated to cirrhosis progression [15]. The contribution of persistant HBV replication to liver cirrhosis and HCC in CHB patients has been determined in several studies [16–18]. Thus, sustained suppression of HBV replication is regarded as critical to reduce liver cirrhosis or HCC progression [19]. Nucleos(t)ide analogues (NAs) have been determined to be highly effective for suppressing HBV replication [20], as well as regression of cirrhosis and reduction of HCC incidence [19, 21–23].

Surgical resection is still regarded as main curative therapy for localized HCC for it may provide probability of disease-free survival in HCC patients [24]. Sustained HBV replication has strong association with HCC recurrence in CHB-related HCC patients after surgery, so anti-HBV treatment is nessarry for CHB-related HCC patients [25]. It has been reported that NAs can provide additional benefits for CHB-related HCC patients receiving local treatment, including hepatic resection [26–28].

Although both monotherapy or combination regimen of NAs have been proved to be effective in CHB-related HCC patients [26–28], it remains to be explored that whether there is any discrepancy among each regimen regarding their potential benefits in CHB-related compensated cirrhosis. Recently, it is reported that nucleutide analogues, rather than nucleuside analogues can induce the expression of IFN-λ [29], which remind us whether there would be any difference regarding the prognosis of CHB-related compensated cirrhosis patients with different NAs regimens. Thus, we designed a retrospective cohort study to explore the potential difference among CHB-related cirrhosis patients with different NAs regimens.

## Methods

### Patients and study design

All CHB patients were diagnosed with compensated liver cirrhosis and received anti-HBV treatment in sixth people’s hospital of Qingdao (Shandong, China). Inclusion criteria were: (1) Diagnosed as HBV infection with compensated liver cirrhosis; (2)Child-Pugh scoring ≤9; (3)Valid clinical characteristics and laboratory outcomes. The exclusion criteria were: (1)Hepatocellular carcinoma; (2)HCV and HDV co-infection; (3)Alcoholic hepatic diseases; (4)Schistosomiasis; (5)Invalid clinical characteristics and laboratory outcomes; (6)Anti-virus regimen switching from non-TDF containing to TDF containing vice versa or received interferon treatment. Three hundred forty-seven patients were recruited into this retrospective study and 94 patients were excluded due to HCV co-infection (*n* = 12) and HDV co-infection (*n* = 11), alcoholic hepatic diseases (*n* = 6), schistosomiasis (*n* = 4), invalid data (n = 6), regimen switching due to virologic breakthrough (*n* = 36), interferon treatment (*n* = 10) and lost (*n* = 20). The final patients included in this study were 242.

This study was conducted under compliance with the Declaration of Helsinki and were approved by *the Human Ethics Committee of sixth people’s hospital of Qingdao.*

### Diagnosis

All patients were histologically confirmed with cirrhosis using specimen from liver biopsy or contrast-enhanced CT. HBV infection were diagnosed with positive serum viral marker and elevated serum HBV-DNA level (> 1000 copies/mL during two consecutive detection). Quantification of serum HBV DNA was measured by real-time quantitative PCR assay with Roche LightCycler (Roche Diagnostics, Basel, Switzerland) and suitable 2reagents (PG Biotech, Shenzhen, China), of which the lower limit of quantification is 1000 copies/mL and the linear range was between 1120 and 6.69 log copies/mL. Contrast-enhanced CT, ultrasonography or liver biopsy were conducted to screen HCC recurrence during follow-up. Child-Pugh scoring was applied for consideration of prognosis as previously reported [30].

### HBV treatment

TDF-containing regimen included TDF monotherapy (*n* = 13) or combined with lamivudine (LAM) (*n* = 95) and entecavir (ETV) (*n* = 19). Non-TDF containing regimen included LAM (*n* = 42), ETV (*n* = 61) or telbivudine (LdT) (*n* = 12) monotherapy. The dosage of NAs in all patients were 300 mg per day for TDF, 100 mg per day for LAM, 0.5 mg per day for ETV and 600 mg per day for LdT. In non-TDF containing group, 11 patients receiving LAM and 12 patients receiving LdT were switch to ETV due to virologic breakthrough.

### Statistics

Continuous variables were expressed as mean ± SD with normal distribution and median (range) without normal distribution. The comparison of continuous variables with or without normal distribution was analyzed with Student *t* test and Wilcoxon rank test, respectively. Chi-square and Fisher’s test were applicated for analysis of categorical variables. *p* < 0.05 was regarded as statistically significant. The univariate analysis were conducted through Kaplan-Meier statistics and Log-rank test. Multivariate analysis was assessed with Cox regression test. Variables with *p* < 0.05 were employed into the Cox regression model. *p* < 0.05 was considered as statistically significant. Statistics analysis was conducted with SPSS (version 16.0, SPSS Inc., Chicago, IL, USA) software package. Figures were made with GraphPad Prism 5 software.

## Results

### The baseline characteristics

242 CHB patients with compensated cirrhosis were attributed to TDF-containing group (*n* = 127) and non-TDF-containing group (*n* = 115) regarding their anti-virus regimen. The average age of TDF-containing group and non-TDF-containing group were 50 ± 12 and 50 ± 10, respectively. Male patients were predominant in both groups: TDF-containing group (*n* = 114, 89.8%) and non-TDF-containing group (*n* = 98, 85.2%). Median total bilirubin was 16.1 and 14.4 μmol/L in TDF-containing group and non-TDF-containing group, respectively. The percentage of patients with Child-Pugh scoring A was 96.1% (*n* = 122) in TDF-containing group and 95.6% (*n* = 110) in non-TDF-containing group (Table 1).

**Table 1:** Baseline characteristics of CHB-related compensated cirrhosis

	TDF-containing	Non-TDF-containing	*p*-value
	(*n* = 127)	(*n* = 115)	
Age, (mean ± SD)	50 ± 12	50 ± 10	0.62
Gender			0.28
Male	114 (89.8%)	98 (85.2%)	
Female	13 (12.4%)	17 (9.7%)	
HBV DNA (log10 copy/mL, mean ± SD)	3.93 ± 1.31	3.88 ± 1.18	0.76
HBeAg			0.92
Positive	92 (72.4%)	84 (73.0%)	
Negative	35 (27.6%)	31 (27.0%)	
ALT (U/L, mean ± SD)	98.3 ± 23.6	96.3 ± 24.4	0.34
Total bilirubin (μmol/L), median (range)	16.1 (5.7–65.0)	14.4 (5.5–39.8)	0.11
ALB (g/L), median (range)	4.1 (3.7–5.9)	4.0 (2.8–6.0)	0.19
AFP, ng/mL, median (range)	2.91 (1.21–34)	3.11 (1.36–21)	0.06
PLT, 10^9/L, median (range)	197.33 (99–252)	184.31 (108–239)	0.08
Child-Pugh score			0.87
A	122 (96.1%)	110 (95.6%)	
B	5 (3.9%)	5 (4.4%)	

*HBeAg* hepatitis B e antigen, *SD* standard deviation

No significant difference of virological characteristics between two groups (Table 1). Serum HBV-DNA level in TDF-containing group and non-TDF-containing group were 3.93 ± 1.31 (log10 copy/mL, mean ± SD) and 3.88 ± 1.18 (log10 copy/mL, mean ± SD), respectively. The percentage of HBe antigen were 72.4% (*n* = 92) in TDF-containing group and 73.0% (*n* = 84) in non-TDF-containing group. 37% (*n* = 47) patients in TDF-containing group and 26.9% (*n* = 31) in non-TDF containing group were HBe antibody positive. Thirteen patients in TDF-containing group and 9 patients in non-TDF containing group were HBsAg negative.

### Virological, serological and biochemical response

All patients achieved virological response by 48 weeks after NAs regimens treatment (HBV DNA < 1000 copies/mL). Eleven patients receiving LAM and 12 patients receiving Ldt in non-TDF-containing group were switched to receiving ETV by 24 weeks. Thirty-two patients in TDF-containing group and 16 patients in non-TDF-containing group experienced virological breakthrough due to poor compliance (HBV DNA > 1000 copies/mL). 64.1% patients (*n* = 59) achieved HBeAg seroconversion in TDF-containing group and 51.2% patients (*n* = 43) in non-TDF containing group. All patients achieved ALT normalization by 96 weeks.

### The development of HCC

**Fig. 1:**
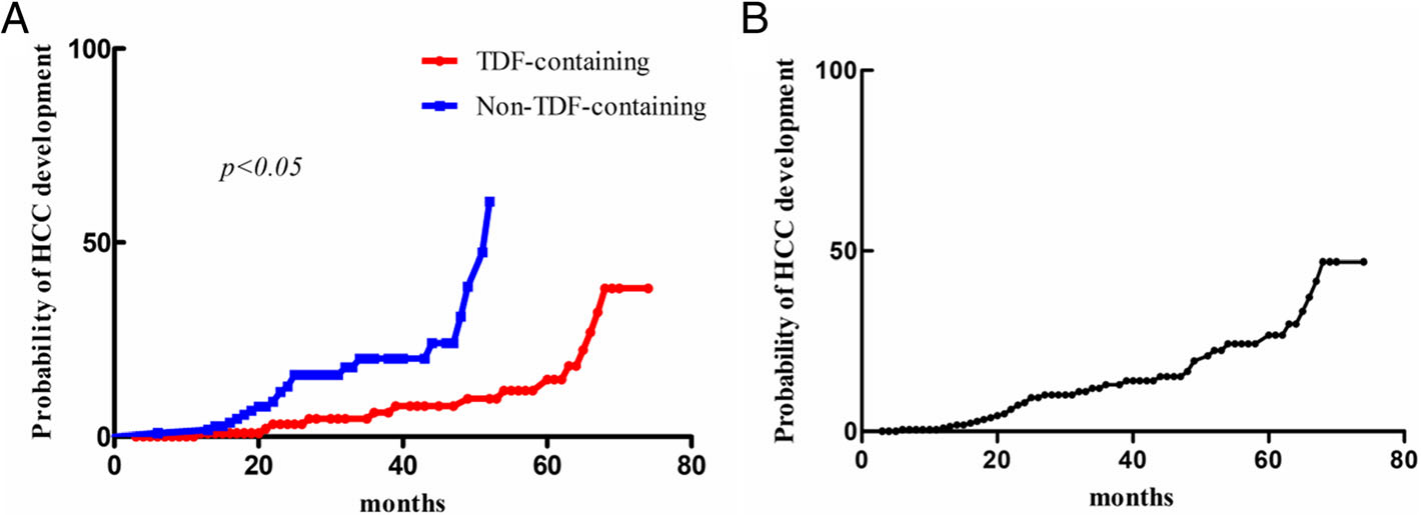
The cumulative probability of HCC development. **a** the comparison of cumulative HCC development probability between TDF-containing group (red) and non-TDF-containing group (blue). X-axis represented time (month), Y-axis represented probability of HCC development. **b** the cumulative probability of HCC development

Overall, 14.5% patients (*n* = 35) developed HCC during follow up. 11.0% patients (*n* = 14) in TDF-containing and 18.3% patients (*n* = 21) in non-TDF-containing group developed HCC. 5-year cumulative rate of HCC development in all patients were 26.7%. The cumulative probability of HCC development in TDF-containing group was significantly lower than it in non-TDF-containing group (hazard ratio, 0.18; 95% confidence interval range, 0.07–0.45, *p* < 0.05) (Fig. 1). In order to identify the potential factors related to the probability of HCC development, we also conduct univariant and multi-variant analysis to investigate the association between baseline characteristics and HCC development. Univariant and multi-variant analysis indicated that TDF-containing regimen treatment was independently associated with lower HCC development rate (hazard ratio, 0.18; 95% confidence interval range, 0.07–0.45, *p* < 0.05) (Table 2).

**Table 2:** Univariate and multivariate analysis of baseline characteristics with HCC development

	Univariant analysis	*p*-value	Multivariant analysis	*p*-value
	HR (95% CI)		HR (95% CI)	
Age	1.00 (0.99–1.01)	NS		
Gender:male/female	3.11 (0.99–9.72)	NS		
Child-Pugh score: A/B	0.48 (0.21–1.10)	NS		
ADV-containing/Non-ADV-treatment	0.18 (0.09–0.43)	< 0.05	0.18 (0.07–0.45)	< 0.05
Total bilirubin: < 24/≥ 24 (μmol/L)	0.52 (0.19–1.41)	NS		
HBV DNA: < 4/≥ 4 (log10 copies/mL)	0.85 (0.41–1.76)	NS		
HBeAg: Positive/negative	1.16 (0.54–2.50)	NS		

*HBeAg* hepatitis B e antigen

## Discussion

Our study demonstrated that anti-virus regimen containing TDF provide a lower probability of HCC development than regimen without TDF in CHB related compensated cirrhosis patients. It has been determined that sustained suppression of HBV replication is fundamental for CHB patients and delays hepatic diseases progression to end stage liver diseases, such as decompensated cirrhosis and HCC [31, 32]. However, regarding the access to anti-virus agents, what kind of treatment is suitable for patients with cirrhosis is not determined. Our study provided evidence for appropriated anti-virus treatment to management of CHB related compensated cirrhosis patients.

Recent studies revealed that nucleutide analogue rather than nucleoside analogue provide additional effect to induce expression of interferon-λ3 [29], since interferon-λ3 has been demonstrated to be involved in modulation of imunity during virus infection or autoimune diseases [33]. Inflammation is determined to have a strong association with carcinogenesis and recurrence of HCC [34]. In our study cumulative probability of HCC development in TDF containing group is significantly lower than non-TDF containing group, which might be caused by hypothesis above. But the mechanism requires further clinical evidence.

The main flaw in our study is the limitation to serum HBV marker quantification, which might reveal the potential mechanism of our outcomes. The association between nucleotide analogues treatment and HBsAg reduction have been proved [29], as well as the relationship between HCC and HBsAg [35]. As a retrospective study, the bias in data collection and poor compliance of patients also limit further analysis for majority of patients included in our study received anti-virus treatment outside of hospital. Thus, a prospective randomized clinical trial to compare anti-virus regimens can provide a better analysis and solid evidence.

## Conclusion

Both anti-virus regimens provided benefit for the prognosis of CHB related compensated cirrhosis patients. Regimen containing TDF, alone or in combination with other agents, lower the incidence of HCC development in CHB patients with compensated cirrhosis.

## Abbreviations

ALT: alanine aminotransferase
CHB: chronic hepatits B
ETV: entecavir
HBeAg: hepatitis B e antigen
HBsAg: hepatits B surface antigen
HBV: hepatitis B virus
HCC: hepatocellular carcinoma
LAM: lamivudine
LdT: telbivudine
NAs: Nucleus(t)ide analogues
SD: standard deviation
TDF: Tenofovir disoproxil fumarate
